# Metal nanoparticles assisted revival of Streptomycin against MDRS *Staphylococcus aureus*

**DOI:** 10.1371/journal.pone.0264588

**Published:** 2022-03-24

**Authors:** Nadia Ghaffar, Sumera Javad, Muhammad Akhyar Farrukh, Anis Ali Shah, Mansour K. Gatasheh, Bander M. A. AL-Munqedhi, Ozair Chaudhry

**Affiliations:** 1 Department of Botany, Lahore College for Women University, Lahore, Pakistan; 2 Department of Chemistry, Forman Christian College (A Chartered University), Lahore, Pakistan; 3 Department of Botany, University of Education, Lahore, Pakistan; 4 Department of Biochemistry, College of Science, King Saud University, Riyadh, Saudi Arabia; 5 Department Botony and Microbiology, College of Science, King Saud University, Riyadh, Saudi Arabia; 6 Ontario Institute of Agrologist, Ontario, Canada; King Abdulaziz University, SAUDI ARABIA

## Abstract

The ability of microorganisms to generate resistance outcompetes with the generation of new and efficient antibiotics. Therefore, it is critically required to develop novel antibiotic agents and treatments to control bacterial infections. Green synthesized metallic and metal oxide nanoparticles are considered as the potential means to target bacteria as an alternative to antibiotics. Nanoconjugates have also attracted attention because of their increased biological activity as compared to free antibiotics. In the present investigation, silver nanoparticles (AgNPs), zinc oxide nanoparticles (ZnO NPs), copper oxide nanoparticles (CuO NPs), and iron oxide nanoparticles (FeO NPs) have been synthesized by using leaf extract of *Ricinus communis*. Characterization of nanoparticles was done by using UV–Vis Spectroscopy, Fourier Transform Infrared Spectroscopy, Scanning Electron Microscopy, Energy Dispersive X-Ray Analyzer, X-ray Diffraction Analysis, and Dynamic Light Scattering Particle Size Analyzer. Interestingly, Streptomycin when combined with AgNPs, ZnO NPs, CuO NPs, and FeO NPs showed enhanced antibacterial activity against clinical isolates of *S*. *aureus* which suggested synergism between the nanoparticles and antibiotics. The highest enhanced antibacterial potential of Streptomycin was observed in conjugation with ZnO NPs (11 ± 0.5 mm) against *S*. *aureus*. Minimum inhibitory concentration of conjugates of AgNPs, ZnO NPs, CuO NPs, and FeO NPs with streptomycin against *S*. *aureus* was found to be 3.12, 2.5,10, and 12.5 μg/mL respectively. The considerable point of the present investigation is that *S*. *aureus*, which was resistant to streptomycin becomes highly susceptible to the same antibiotic when combined with nanoparticles. This particular observation opens up windows to mitigate the current crisis due to antibiotic resistance to combat antimicrobial infections efficiently.

## Introduction

The emergence and spread of antimicrobial-resistance is imposing a global challenge. Drug resistance is a crucial issue that is emerging from decades as an inevitable and natural process because of misuse and overutilizing the antimicrobial drugs. One of the most common causes of deaths worldwide is occurance of infectious diseases caused by pathogenic bacteria and it’s a constant health risk globally [1]. Nearly everyone in the health sector is affected by the infections caused by drug-resistant bacteria. The UK Government-commissioned Neil report predicted that without urgent action 10 million people a year will die from drug-resistant infections by 2050 [2].

*S*. *aureus* is a gram-positive bacterium causing several health problems, ranging from common skin infections to life-threatening illnesses including abscess infection, pneumonia, pseudomembrane enteritis, osteomyelitis, and sepsis [3,4]. This microbe has developed resistnace against a number of antibiotics and has emerged as a threat to human life. It has many characteristics that make it complicated to treat like gliding mobility, slime production, staphyloxanthin pigment production, and most alarming is biofilm formation [5,6]. It was reported earlier that 90% of *S*. *aureus* are resistant against penicillin while 70–80% are resistant against Methicillin. The present situation of hospital, livestock, and community-associated resistant strains is even more threatening [7,8].

There are numer of targets of antibiotics in *S*. *aureus* including cell envelope, ribosomes, and nucleic acid. But this cunning microbe has developed various pathways to escape from the antibiotic’s attack. It includes a modified way of protein synthesis which is not ribosome dependent. Furthermore, it can modify attacking drug or binding site of drug with the help of its enzymes. There are also reports that these bacteria enhance drug efflux mechanism, inactivate drug, or displace the drug, etc [9]. Multidrug-resistant strains (MDRS) of bacteria are defiant of even 3^rd^ generation antibiotics. In *S*. *aureus*, there has been widespread resistance reported [9–11].

The main reasons for increased resistance cases of microbes are misuse of antibiotics including self-medication, overuse of antibiotics, and higher transmission rates due to population density [12,13]. To combat the increased microbial infections and to increase the health standards of a common man, more antibiotics/antimicrobial agents are required. But the discovery of a new antibiotic or antimicrobial agent, its biosafety tests, and its release for safe human consumption takes decades [14]. Strategies to preserve antibiotic effectiveness by enhancing the activity of already approved antibiotics can be a solution in such a drastic situation. One of the alternatives to fight against multi-drug resistant organisms is the use of nanoantibiotics and nanomaterials with antimicrobial properties [15]. Nanotechnology is proving its strength in this field where metal and metal oxide nanoparticles like silver nanoparticles (AgNPs), zinc oxide nanoparticles (ZnO NPs), copper oxide nanoparticles (CuO NPs), and Iron oxide nanoparticles (FeO NPs) are bio-safe, less toxic, and biocompatible [3,16,17]. But they are toxic against many pathogenic bacterial strains even at lower concentrations, particularly highly toxic to gram-positive bacteria like *S*. *aureus* [18–21].

Nanoparticles contain distinctive physicochemical properties which can introduce new modes of antibacterial action. Nanoparticles affect microbes differently and their activity depends upon their shape and size. Rod-like ZnO nanoparticles have higher antibacterial activity as compared to plate-like nanoparticles [22,23]. Cubical silver nanoparticles have shown higher antibacterial activity as compared to round silver nanoparticles [24]. Edges and vertexes on the surface of nanoparticles help them to enter into the cell walls of microbes. NPs with larger surface areas release more reactive oxygen species from the bacterial cells, exposing them to higher stress. Moreover, there is a very lesser rate of resistance development within microbes against nanoparticles [25].

Due to their antibacterial and antiviral activity, nanoparticles are the most promising antimicrobial nowadays [26,27]. Metal NPs have a generalized mode of action against bacteria, viruses, and fungi. They show a much higher effectiveness to microbial infections regardless the microbial susceptibility to conventional antibiotics [28], so they can be used as a potent antibacterial agent to overcome the problem of resistance development in microbes against conventional antibiotics. Metal NPs have many other advantages besides their antimicrobial properties. These particles can be synthesized by simple, cost-effective, biocompatible, environmentally friendly, and facile methods. Green synthesis of nanoparticles (nanoparticles synthesized by using plant extract and metal salts) with a low range of toxicity has become an attractive area of research for several biomedical applications [29].

Nanoparticles have a multi-level mode of action on bacterial cells and these can affect many microbial metabolic processes. These can interfere with the cell wall and cell membrane, causing their disruption and increasing their cell permeability [30]; so that antibiotics can easily penetrate the bacterial cell and disrupt metabolic pathways. These can interact with microbial DNA and produce reactive oxygen species which damages biomacromolecules [31,32].

Recently, advancements in the synergistic effect of combined formulation of antibiotics with NPs have been reported. Studies focused on the mechanism of action of the NPs-antibiotic combinations have suggested that the enhancement in antibacterial action may be due to chemical interaction between NPs and antibiotics. Augmentation of antibiotics with nanoparticles not only reduces the requirement of a high dosage of medicine but also minimizes its toxicity toward human cells and reinstates its capability to inhibit microbes that have developed resistance [33]. Furthermore, nanoparticles attached with antibiotics have also enhanced the quantity of antibiotics at the interaction point of bacterium to antibiotic and helped in the binding of antibiotics to bacteria, and blocking bacterial efflux pumps which caused increased efficiency of the conjugate. Nanoparticles-antibiotic-complex is supposed to be used as an alternative to resistant microbes. Because nano-conjugates exhibit a notable increase in their biological activity as compared to free antibiotic molecules [34]. It is reported by Nishanthi *et al*., 2019 that streptomycin in combination with AgNPs showed enhanced activity, its activity increased up to 87.5% [35]. Salar *et al*., 2015 studied the enhanced activity of streptomycin (57.98%) in conjugation with nanoparticles [36]. Aremu *et al*., 2021 reported that AgNPs synergistically increase the antibacterial activity of streptomycin (30–52%) when they are applied in combination [37].

The enhancement of the antimicrobial activity due to NPs-antibiotic combinations, would enable us to use the antibiotics that have fallen into disuse because of bacterial resistance issues, providing additional treatment possibilities in the healthcare, veterinary, and agriculture sectors. Thus, nano antibiotics will definitely have a potential impact on social and economic problems, as they may help to mitigate the current crisis of antibiotic resistance.

Therefore, in the present study, AgNPs, ZnO NPs CuO NPs, and FeO NPs were synthesized by using an aqueous extract of *R*. *communis* with metal salts (AgNO_3_, ZnSO_4_, CuSO_4_, FeCl_3_). Synthesized NPs were characterized by using different techniques. NPs then used for the preparation of conjugates with streptomycin. The antibacterial activity of metal salts, plant extracts, metal nanoparticles, and streptomycin-NP combination was evaluated against *S*. *aureus*. Then MIC was calculated to find out the minimum amount of antibiotic-NP conjugate to inhibit the growth of (MDR) *S*. *aureus*.

## Materials and methods

### Materials

Silver nitrate, Zinc sulfate, Copper sulfate and, Iron chloride were purchased from Sigma Aldrich (USA). Streptomycin was also purchased from Sigma Aldrich (USA). All reagents were of analytical grade and used as received. Sterile double distilled water was used for the preparation of solutions, to avoid any photochemical reaction; prepared solutions were kept in dark.

### Pathogenic strain

The bacterial pathogen *S*. *aureus* (clinical isolate, pathogenic) was received from the University of Health Sciences Lahore, Pakistan. The strain was maintained on Mueller Hinton agar slants at 4°C.

### Collection and preparation of plant material

Fresh leaves of *R*. *communis* were collected from Johar Town, Lahore, Pakistan. Just after the collection of plant material, it was washed thoroughly with fresh water, identified from its morphological characters, and then washed with distilled water. Clean plant material was air-dried in the lab, crushed manually, and ground by using an electric grinder to get fine texture powder, sieved through a mesh to get particles of uniform size, and stored in an airtight sterilized container. 50 g of the powdered plant material was weighed accurately and added to 500 mL of distilled water, mixed thoroughly, and subjected to microwave-assisted extraction (power level 600W for 3.5 minutes) in closed vessel type. The extract obtained from MAE was filtered and the filtrate was concentrated in a rotary vacuum until a crude solid extract with a yield of ~15% was achieved. It was saved at 4°C for further analysis. Before using for experimentation 12 g L^-1^of the dried extract solution was prepared in 50 mL of 0.1 M NaOH solution [38].

### Synthesis of nanoparticles

Aqueous solutions (2 mM) of each of the salt i.e., silver nitrate, zinc sulfate, copper sulfate, and iron chloride were prepared. For the synthesis of NPs different volumes of plant extracts (10, 20, 30 mg/mL) were added dropwise in the prepared salt solution by continuous stirring at 80 rpm by using a magnetic stirrer, at room temperature. By varying the ratio of plant extract to a fixed amount of salt solution (10mL of 2mM solution), three types of nanoparticles for each salt were obtained as mentioned in Table 1. The obtained suspensions were centrifuged at 13,000 rpm for 15 min. The supernatant was discarded and pellets containing nanoparticles were washed 3–4 times with deionized water and stored at a cool dry place for further analysis.

**Table 1:** Composition of nanoparticles forming solutions.

Meta salt	Plant extract used	Ratio of salt to plant material	Abbreviated name
Silver nitrate (10mL)	**10 mL**	**1:1**	**AgR**_**1**_
	**20 mL**	**1:2**	**AgR**_**2**_
	**30 mL**	**1:3**	**AgR**_**3**_
Zinc sulfate (10mL)	**10 mL**	**1:1**	**ZnR**_**1**_
	**20 mL**	**1:2**	**ZnR**_**2**_
	**30 mL**	**1:3**	**ZnR**_**3**_
Copper sulfate (10mL)	**10 mL**	**1:1**	**CuR**_**1**_
	**20 mL**	**1:2**	**CuR**_**2**_
	**30 mL**	**1:3**	**CuR**_**3**_
Iron chloride (10mL)	**10 mL**	**1:1**	**FeR**_**1**_
	**20 mL**	**1:2**	**FeR**_**2**_
	**30 mL**	**1:3**	**FeR**_**3**_

### Conjugation of antibiotic with nanoparticles

Conjugates were prepared to evaluate the antibacterial activity of Streptomycin in combination with all types of green synthesized nanoparticles against *S*. *aureus*. Nanoparticles synthesized as per previous section, depicting the highest antibacterial activity, were selected for the preparation of conjugates with the antibiotic.

For conjugation of antibiotic with nanoparticles, 1 mg/mL of prepared NPs were mixed with 1 mg/mL of antibiotic (Streptomycin) (concentration for an antibiotic was selected according to CLSI standard) in 1 mL of phosphate buffer solution. The reacting mixture was incubated for 24 hours and then centrifuged for 10 min at 10,000 rpm. The antibiotic conjugated nanoparticles were obtained in the form of pallets that were washed thrice, dried, and then used for further studies [39].

### Characterization of nanoparticles

#### UV–Visible spectroscopy

UV–Vis spectroscopy is an easily available and most effective technique that allows fast identification of nanoparticles formation. Bio reduction of metal ions in the solution was monitored by UV–Visible spectroscopy (BMS, UV-2600). The mixture of a metal salt with different volumes of plant extract was analyzed for its spectra and ƛ_max_. For this purpose small amount of sample was taken in a quartz cuvette and distilled water was used as a reference, the wavelength was recorded in the range of 200 to 700 nm using a spectrophotometer.

#### Particle size analysis

The particle size of NPs was determined by using BT 90 Nano laser particle size analyzer. For this purpose samples were prepared by dissolving 1 mg of synthesized NPs in 1 mL of distilled water and sonicated properly to dissolve/ suspend the particles. Usually, a suspension is formed which was shaken vigorously before analysis. Prepared samples were subjected to a particle size analyzer and average particle size was determined.

#### Fourier transform infrared (FTIR) spectroscopy

FTIR plays an important role in identifying surface absorbents present in synthesized nanoparticles. For FTIR analysis, samples were prepared by using desired material and potassium bromide powder. Pallets were prepared and analyzed in an IRT racer-100 FTIR spectrophotometer and the spectrum was saved at a resolution of 4 cm^−1^ in the range of 650–4000 cm^−1^. The various modes of vibrations were identified and assigned to determine the different possible functional groups present in the synthesized nanoparticles which are responsible for capping and efficient stabilization of the synthesized nanoparticles.

#### Scanning electron microscopy

Scanning electron microscopy was used to identify the surface morphology of prepared NPs. For SEM analysis, slides were prepared by using a minute amount of NPs and forming a thin smear on the slides. Slides were dried and coated with a slight layer of platinum to make them conductive. Prepared slides were subjected to SEM (ZEISS-EVO/LS10) at 20 kV voltage for characterization. SEM graphs were collected at different magnifications.

#### Energy-dispersive X-ray

The qualitative and quantitative identification of the elemental composition of metal and metal oxide NPs was analyzed by using SEM (ZEISS-EVO/LS10) at 20 kV voltage.

#### X-ray diffraction analysis

To study the crystalline structure and average crystallite size of the synthesized nanoparticles, X-ray diffraction analysis was performed. X-ray powder diffraction patterns were obtained with Cu-Ka radiation using a Shimadzu XRD (model 6000) diffractometer (Japan) equipped with a graphite monochromator. Properly dried NPs in powdered form (50 mg) were utilized for analysis. XRD measurements on a film of green synthesized NPs were performed using a step scanning program with 0.02° per step and acquisition time of 5 seconds per step at 2 theta. The XRD data were analyzed by using a standard powder diffraction card (JCPDS-ICSD (Joint Committee on Powder Diffraction Standards-International Center for Diffraction Data)) for the identification of the crystalline phases. The average crystallite size of NPs was estimated by using the Scherrer equation

D=Kλ/βcosθ

Where, D is the crystallite size (nm), K = 0.9 (Scherrer constant) λ = 0.15406 (X-ray wavelength), β = FWHM (full width at half maximum of the diffraction peak) and θ = peak position.

#### Evaluation of antibacterial activity

Antibacterial activity of plant extracts, antibiotic, nanoparticles, and conjugates of antibiotic and nanoparticles was checked and compared against resistant species of *S*. *aureus* by using agar well diffusion assay and MIC was determined by broth dilution method.

#### Preparation of active cultures

Blood agar plates were prepared and a loop full of cells from the stock cultures were transferred to plates for preparation of active culture for experimentation. Plates were then incubated without agitation for 24 hours at 37°C. Fresh cultures were utilized for further experimentation. This process was repeated every time before starting a new experiment.

#### Preparation of agar medium

The medium was prepared by adding 38 g of agar medium in 1 liter of purified water. It was mixed thoroughly and boiled for 1 minute to completely dissolve the medium and the mouth of the flask was covered with foil paper. Then the medium was autoclaved for 15 min at 121°C. After autoclaving, the flask was placed in a water bath to cool, and the then molten cooled medium was poured in sterile Petri plates on a level, horizontal surface to give uniform depth under aseptic conditions and left for 24 hours at 37°C to check any contamination. The plates were poured to a depth of 4 mm.

#### Preparation of McFarland standard

For the preparation of McFarland standard 1% solution of anhydrous barium chloride (BaCl_2_) and 1% solution of sulfuric acid (H_2_SO_4_) were prepared.

For preparing 0.5 McFarland turbidity standards, 0.05 mL of 1% solution of BaCl_2_ and 9.95 mL of H_2_SO_4_ were mixed carefully in a screw-capped test tube to form a turbid suspension. The resulting suspension was rapped by using foil paper and stored at room temperature. Before every use, the tube was shaken vigorously [40].

#### Preparation of inoculum

The inoculum was prepared by suspending isolated colonies from an 18–24 hour plate in saline solution to turbidity matching a 0.5 McFarland standard. The inoculum suspension was used within 15 min of preparation. A sterile cotton swab was dipped into the suspension and pressed firmly on the inside of the tube to remove excess liquid. The dried surface of the appropriate agar plate is inoculated by streaking the entire surface and repeating it twice; rotating the plate 60 degrees each time to obtain an even distribution of the inoculum.

#### Antibacterial activity of the green synthesized nanoparticles

Prepared Petri dishes were brought in a laminar flow transfer cabinet and stainless steel cork borer was used to form wells of 10 mm diameter on agar plates. 1 mg/mL solution of NPs was prepared using sterile water and sonicated properly. Prepared solutions of all NPs were further diluted to get an exact concentration of 100 μg, 50 μg, and 25 μg/mL. 100 μL of prepared plant extract, metal salt solution, and prepared solutions of nanoparticles (100 μg, 50 μg, 25 μg/mL) were loaded in different wells in the agar plate. The plates were allowed to remain undisturbed for 1 hour to ensure even diffusion of samples into the agar. The plates were incubated for 24 hours at 37°C. The inhibition zones formed around the wells were measured in millimeters [41].

#### Evaluation of the synergistic effect of antibiotic in combination with NPs

Concentrations of NPs which provided the highest antibacterial activity were selected for conjugation with streptomycin. The combined formulation of streptomycin with prepared NPs was also inoculated in the prepared wells to assess the enhancement of the antibacterial activity of an antibiotic. The prepared formulation (25 μg/mL) of NPs with antibiotic (Streptomycin) were added to the wells and plates were incubated at 35°C for 24-48h. The individual antibiotic (streptomycin, pure standard) was also used to compare its antibacterial activity with its respective conjugates with various NPs. The inhibition zones formed around the wells were measured in millimeters with the help of the inhibition zone measurement scale [35].

#### Determination of minimum inhibitory concentration (MIC) of conjugates

MH broth was prepared by dissolving 21 g of MH broth in 1 liter of distilled water. It was mixed carefully by continuous agitation and heating. It was boiled for 1 minute until it was dissolved completely and then 1 mL of MH broth was poured in each sterile test tube, that were sterilized in an autoclave at 121°C for 15 minutes. The prepared test tubes were cooled down and used for experimentation.

#### MIC of conjugates

The minimum concentration of any material required to inhibit the growth of particular microorganisms is known as minimum inhibitory concentration (MIC). The MIC of antibiotic in combination with NPs was assessed according to the protocol of the Clinical Laboratory Standards Institute 2006. 1 mg/mL solutions of all the conjugates were prepared, further dilutions were made by using distilled water. From an overnight culture, 4–5 isolated colonies were selected and diluted in broth to turbidity comparable to that of a 0.5 McFarland turbidity standard (approximately 1.0 ×10^8^ CFU/mL). The prepared suspension was further diluted at 1:100 (10^6^ CFU/mL) by using distilled water, 0.85% saline, or broth. Sterile test tubes, containing 1 mL of MH broth were taken and prepared formulations were added in tube 1 and 2 that was followed by serial dilution to get an exact concentration of 12.5 μg/mL, 6.25 μg/mL, 3.12 μg/mL, 1.56 μg/mL, 0.78 μg/mL, 0.39 μg/mL and one test tube was taken as control. Then, freshly prepared bacterial suspension was inoculated in all the prepared test tubes including the control. All tubes were incubated in an ambient air incubator at 35–37°C for 24 hours. MIC was determined by visual observation of growth. The minimum concentration of the extract that showed no detectable growth was taken as the minimum inhibitory concentration [41].

#### Assessment of fold area inhibition measurement

Evaluation of increase in fold area was achieved by calculating the mean surface area of zone of inhibition by antibiotic. The given equation was used to calculate the percentage of fold area inhibition for streptomycin.


$$\%\mathrm{Fold}\;\mathrm{increase}=\frac{(\mathrm{SM}+\mathrm{NPs})-\mathrm{SM}\times 100}{\mathrm{SM}}$$

Where,

SM: inhibition zone by streptomycin alone

SM+NP: inhibition zone by streptomycin + green synthesized metal NPs [35].

#### Determination of the effect of conjugates on leakage of membrane

DNS and Bradford’s method were employed to examine the effect of conjugates on leakage of reducing sugars and proteins through the membrane. For this purpose, *S*. *aureus* was grown overnight and 2 mL sample was taken from each culture in test tubes separately and labeled as a 0-hour sample. 1 mL of prepared solutions (1 mg/mL) of NPs + AB was added in culture tubes and incubated at 37°C at 200 rpm. After 2, 4, and 6 hours, the sample was withdrawn and centrifuged at 10,000 rpm for 5min. Immediately, supernatant from the sample was preserved at −30°C, and pallets were discarded. Then the supernatant was examined to determine the concentration of reducing sugar and proteins [41].

### Statistical analysis

SPSS software was used to analyse the significance of the data at 5% level of significance, whereas means were compared by using one way of ANOVA (Analysis of variance). Duncan’s multiple range test was used as Post hoc test and 3 replicates were used for each measurement.

## Results and discussion

### Green synthesis of metal and metal oxide NPs

**Fig 1:**
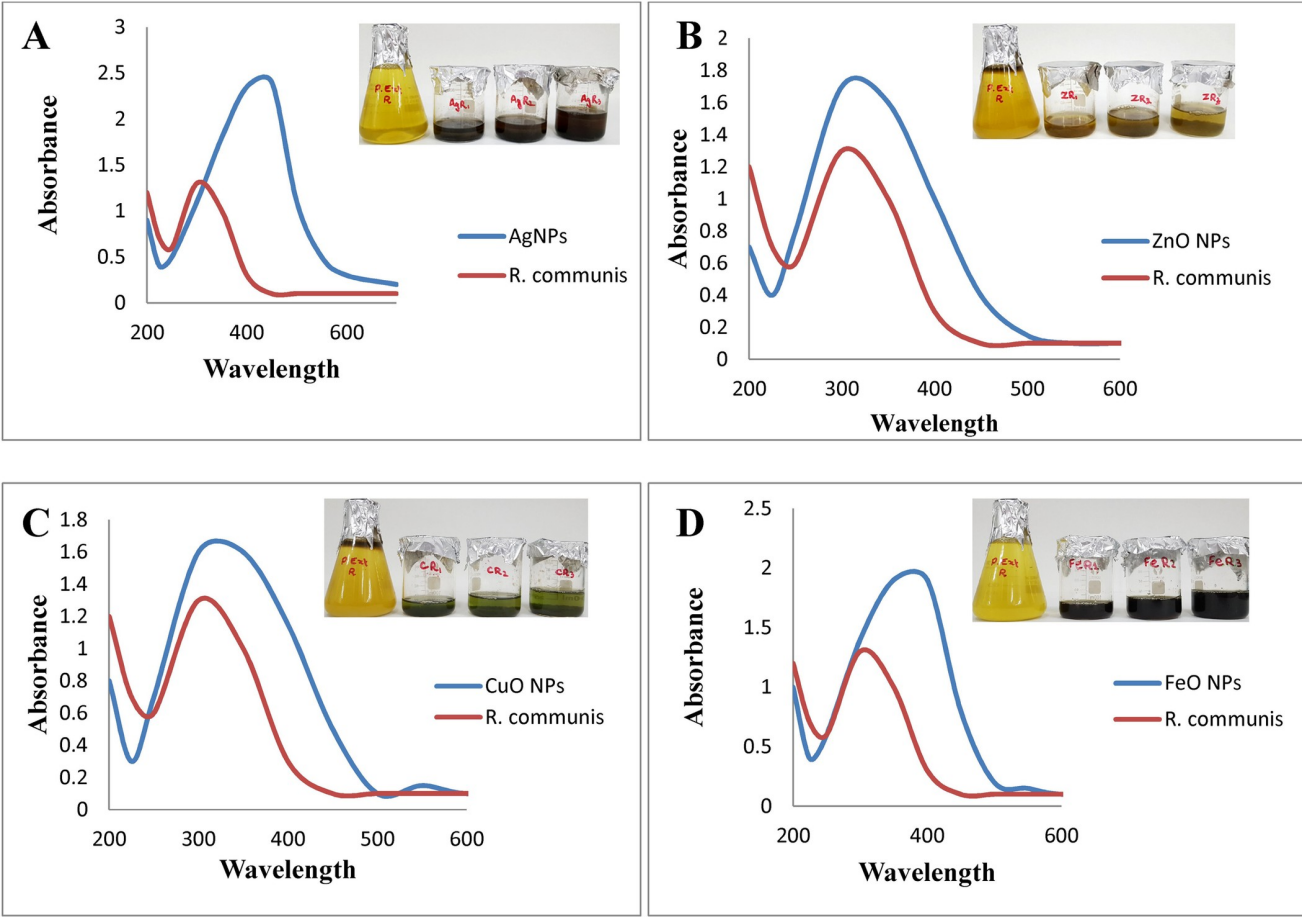
UV–Vis spectrum of *R*. *communis* and (A) AgNPs (B) ZnO NPs (C) CuO NPs and (D) FeO NPs, synthesized by using 10 mL of 2 mM salt solution with 30 mL of plant extract.

In the present investigation, an effort has been made to evaluate the potential of aqueous extract of *R*. *communis* to reduce metal salts that result in the formation of nanoparticles. When different volumes (10, 20, 30 mg/mL) of aqueous extract of *R*. *communis* were added dropwise in prepared solutions (2 mM) of AgNO_3_, ZnSO_4,_ CuSO_4,_ and FeCl_3_ separately at ambient temperature, it led to the synthesis of nanoparticles. A gradual color change was observed from light green to brown, brownish-green, dark green, and dark blackish-brown for silver, zinc, copper, and iron salts respectively which indicated the formation of NPs. By varying the volumes (10, 20, 30 mg/mL) of plant extract to react with a fixed volume (10 mL) of 2 mM salt solution, a slight color difference was observed. 2mM salt solution with 30 mg/mL of plant extract exhibited a more intense change in color as compared to other concentrations (Fig 1A–1D).

The color change is a very good indicator of NPs synthesis [42]. The color change is due to the collective vibrations of the charged particles present on the surface of nanoparticles and the resonance. NPs need reducing agents and stabilizers for their synthesis and to prevent their aggregates formation after synthesis [21]. The phytochemicals present in plant extracts have reducing potential, due to which they are responsible for NPs formations. Plants like *R*. *Communis* have several phenolic compounds with evident antioxidant activity due to the availability of H^+^ ions. These ions are also responsible for the reduction of metal ions into nanoparticles [43,44]. Green synthesis of NPs is also well-thought-out as less toxic to the environment in comparison to chemical synthesis. Synthesis of NPs by visual detection is also explained by Ahmad and Divya [45]. Amutha and Sridhar also confirmed the green synthesis of NPs by using plant extract and salt solution [46].

### UV–Visible spectroscopy

UV–Vis spectroscopy is one of the well-known techniques that are widely used for the characterization of synthesized nanoparticles. It is the simplest way to confirm the formation of nanoparticles. In the present study, AgNPs, ZnO NPs, CuO NPs, and FeO NPs showed maximum absorption at 430, 303, 330, and 404 nm respectively (Fig 1A–1D). UV-Visible spectroscopy (UV-Vis) measures the extinction (scatter + absorption) of light passing through a sample. Due to the unique optical properties of NPs, their UV-visible spectra are sensitive to concentration, shape, size, and refractive indices near the surface of NPs, which makes UV-Vis a valuable tool for studying, identifying, and characterizing nanomaterials [47].

Nanoparticles synthesized by using metal precursors contain free electrons, and the combined vibration of these electrons in resonance with light waves provides surface plasmon resonance (SPR) absorption bands. As nanoparticles diameter increases, the peak wavelength also increases. The absorbance spectrum shifts considerably into the far-red region of the spectrum for uneven-shaped particles in comparison to spherical nanoparticles of the same diameter. An increase in the intensity of the absorption peak was observed by increasing the reaction time. A single prominent peak indicates the presence of uniform size NPs. These results are correlated with the findings of Selvaraj et al., and Amutha and Sridhar [33,46].

### Dynamic light scattering (DLS)

**Fig 2:**
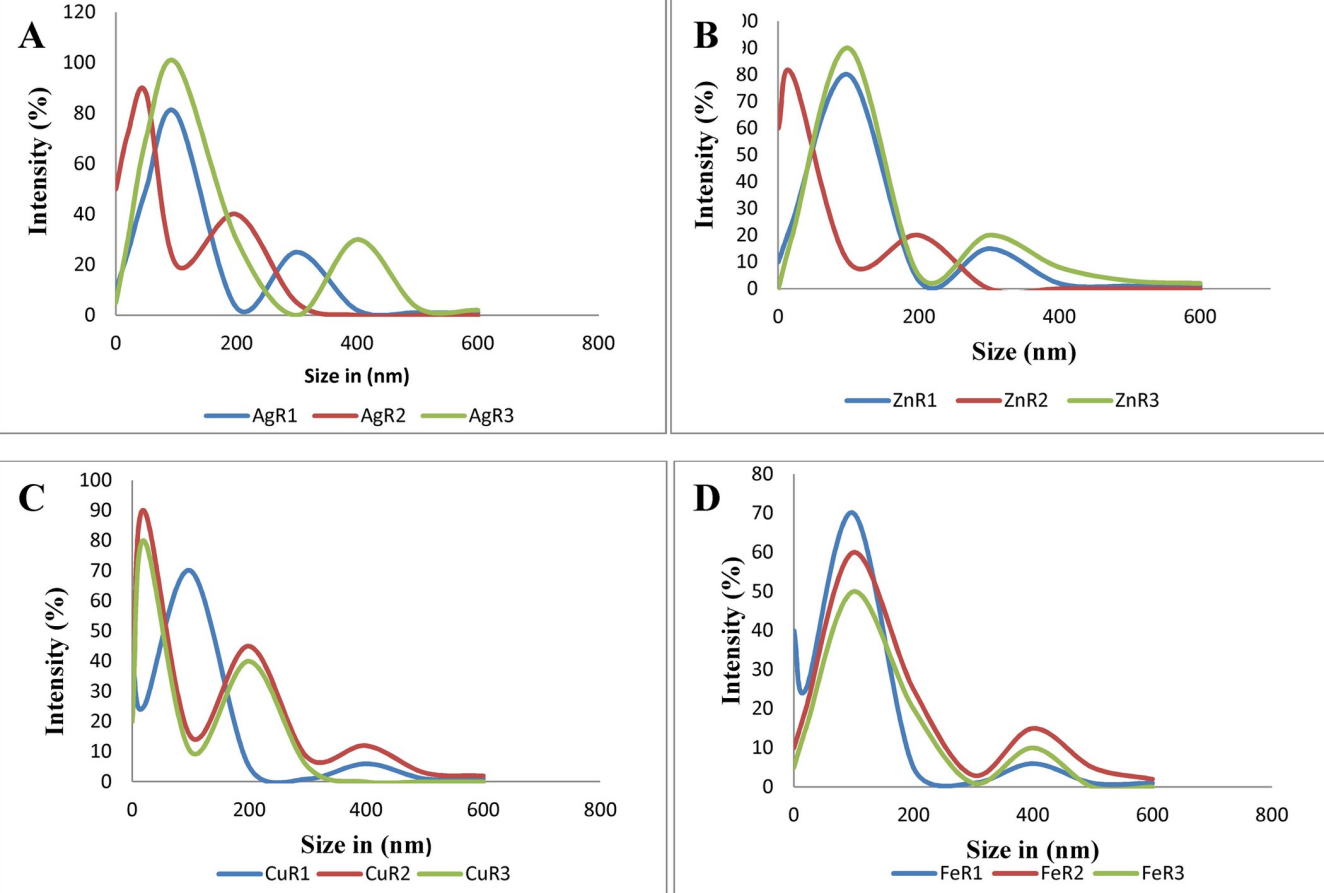
DLS results of (A) AgNPs synthesized by using 10 mL of 2 mM AgNO_3_ solution with 10, 20 and, 30 mL of *R*. *communis* extract (B) ZnO NPs synthesized by using 10 mL of 2 mM ZnSO_4_ solution with 10, 20 and, 30 mL of *R*. *communis* extract (C) CuO NPs synthesized by using 10 mL of 2 mM CuSO_4_ solution with 10, 20 and, 30 mL of *R*. *communis* extract (D) FeO NPs synthesized by using 10 mL of 2 mM FeCl_3_ solution with 10, 20 and, 30 mL of *R*. *communis* extract.

DLS is one of the most practical techniques used for the identification of the size of nanoparticles. It provides information about average particle size distribution. This technique is very fast, sensitive, and can estimate the size of a particle on both nano and macro scales. Average particle size distribution of AgNPs (AgR_1,_ AgR_2,_ AgR_3_), ZnO NPs (ZnR_1,_ ZnR_2,_ ZnR_3_), CuO NPs (CuR_1,_ CuR_2,_ CuR_3_) and FeO NPs (FeR_1,_ FeR_2,_ FeR_3_) is presented in the Fig 2A–2D respectively.

The single peak indicates the uniform distribution of nanoparticles [48,49] as it is clear from peaks presented by nanoparticles. It shows that an increase in the concentration of plant extract in the reaction mixture decreased the NPs size and also makes their distribution uniform. When a larger amount of plant extract is used, it carries a higher amount of antioxidants or reducing plant metabolites. Therefore, for a fixed amount of metal ions present in the solution, if we optimize the amount of plant extract, that can determine the size of nanoparticles.

### Electron microscopic analysis

**Fig 3:**
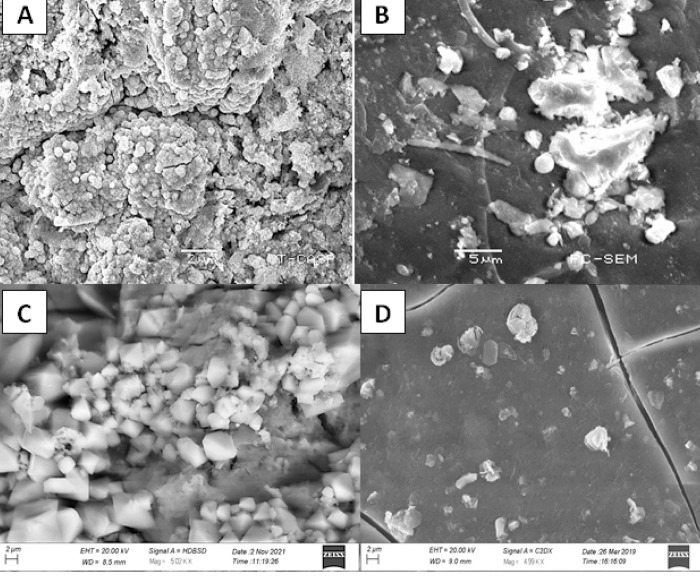
SEM images of (A) AgNPs prepared by using 10 mL (2 mM solution) of AgNO_3_ with 30 mL of *R*. *communis* leaves extract (B) ZnO NPs prepared by using 10 mL (2 mM solution) of ZnSO_4_ with 30 mL of *R*. *communis* leaves extract (C) CuO NPs prepared by using 10 mL (2 mM solution) of CuSO_4_ with 30 mL of *R*. *communis* leaves extract (D) FeO NPs prepared by using 10 mL (2 mM solution) of FeCl_3_ with 30 mL of *R*. *communis* leaves extract.

**Fig 4:**
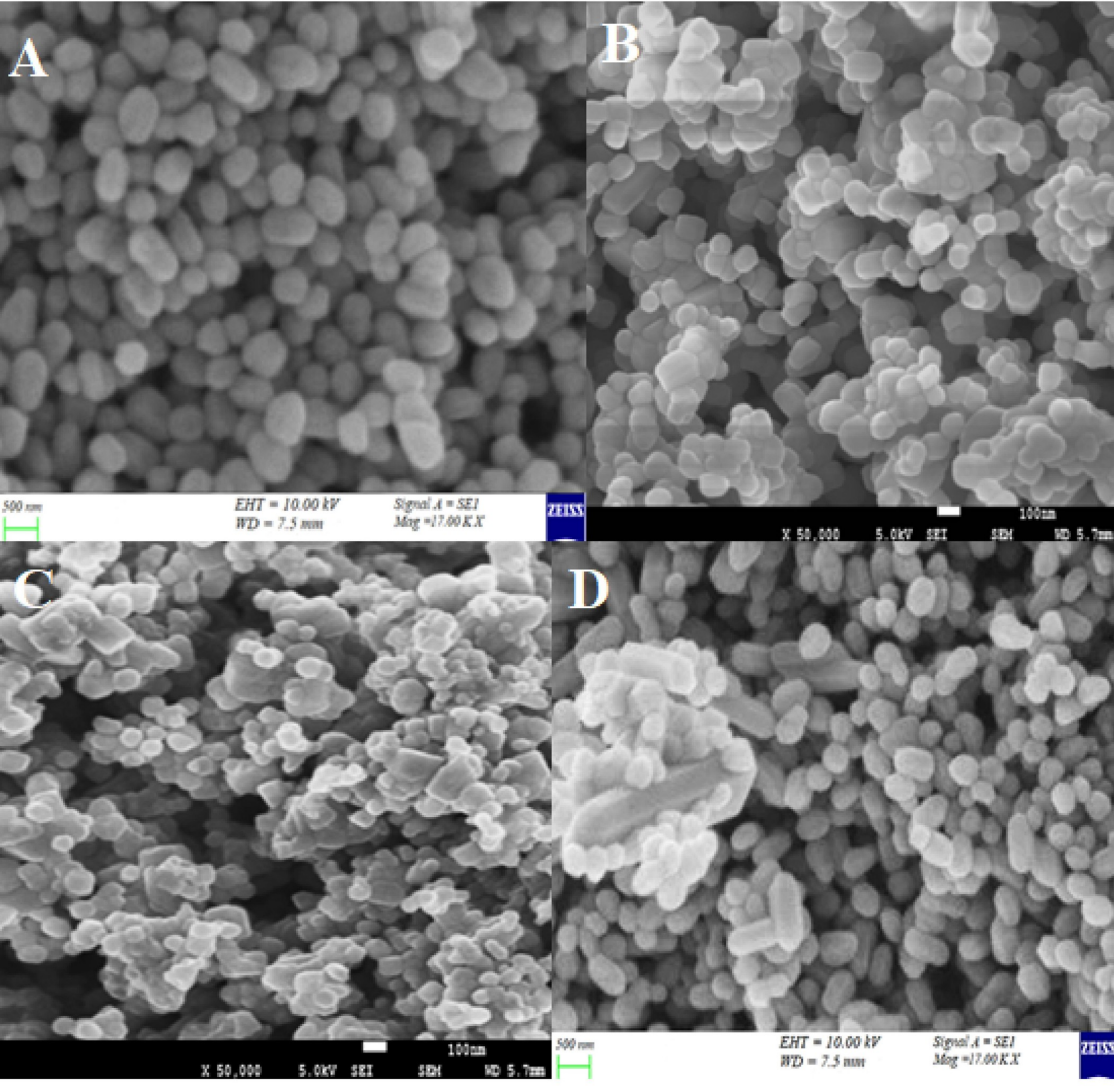
SEM images of conjugated NPs with Stretomycin (A) AgNPs-Stp (B) ZnO NPs-Stp (C) CuO NPs-Stp (D) FeO NPs-Stp.

Surface morphology and topography of nanoparticles and their conjugates with streptomycin, were studied by using a scanning electron microscope (SEM). AgNPs were analyzed by using the micron marker of 02 μm. The SEM images revealed the formation of individual silver nanoparticles, as well as some aggregates, were also seen. Silver nanoparticles were observed in a spherical shape, smooth surface, and closely arranged (Fig 3A). Conjugates of AgNPs with streptomycin presented well-defined spherical and oval shape particles at a micron marker of 500 nm. It showed minimum aggregates, most of the observed particles were independently arranged (Fig 4A).

Zinc oxide nanoparticles were analyzed by using the micron marker of 05 μm at the magnification power of 2400x respectively. SEM images showed that ZnO NPs were not of constant shape as they were giving spherical, hexagonal, square, and triangular morphology. ZnO NPs were found individually and in the form of aggregates while the surface of aggregates was rough (Fig 3B). ZnO NPs-Stp depicted smaller size, smooth-surfaced, closely arranged spherical particles at a micron marker of 500 nm (Fig 4B).

The images for CuO NPs were recorded at a magnification of 5000x by using a micron marker of 02 μm. The topographical view shows that most of the CuO NPs were of a pyramid shape. Some NPs of hexagonal shape were also seen which clustered together and the surface of the aggregates seems to be smooth (Fig 3C). Conjugated CuO NPs with streptomycin were analyzed by using a micron marker of 100 nm. SEM images revealed the formation of smooth surfaced, closely arranged, hexagonal and spherical particles (Fig 4C).

Iron oxide nanoparticles were analyzed by using the micron marker of 02 μm at the magnification power of 5000x. Electron micrograph revealed hexagonal, triangular, and smooth surface particles. SEM images illustrate individual iron oxide nanoparticles as well as many aggregates (Fig 3D). FeO NPs in conjugation with streptomycin showed hexagonal particles at a micron marker of 500 nm. Some hexagonal particles were found individually and in the form of aggregates while some of the particles were just closely arranged (Fig 4D).The presence of different quantities and the nature of capping agents may affect the size and shape of nanoparticles. A thin layer of capping organic material is thought to be present on nanoparticles synthesized by the green method; due to capping organic material, nanoparticles may attain stability in solution. It is very important to study the shape and size of NPs while considering their antimicrobial activity. The antimicrobial activity of NPs is directly related to their shapes and sizes which in turn determines their mode of action and their interaction with the microbe surface [50].

### Energy-dispersive X-ray spectrometer (EDX) analysis

**Fig 5:**
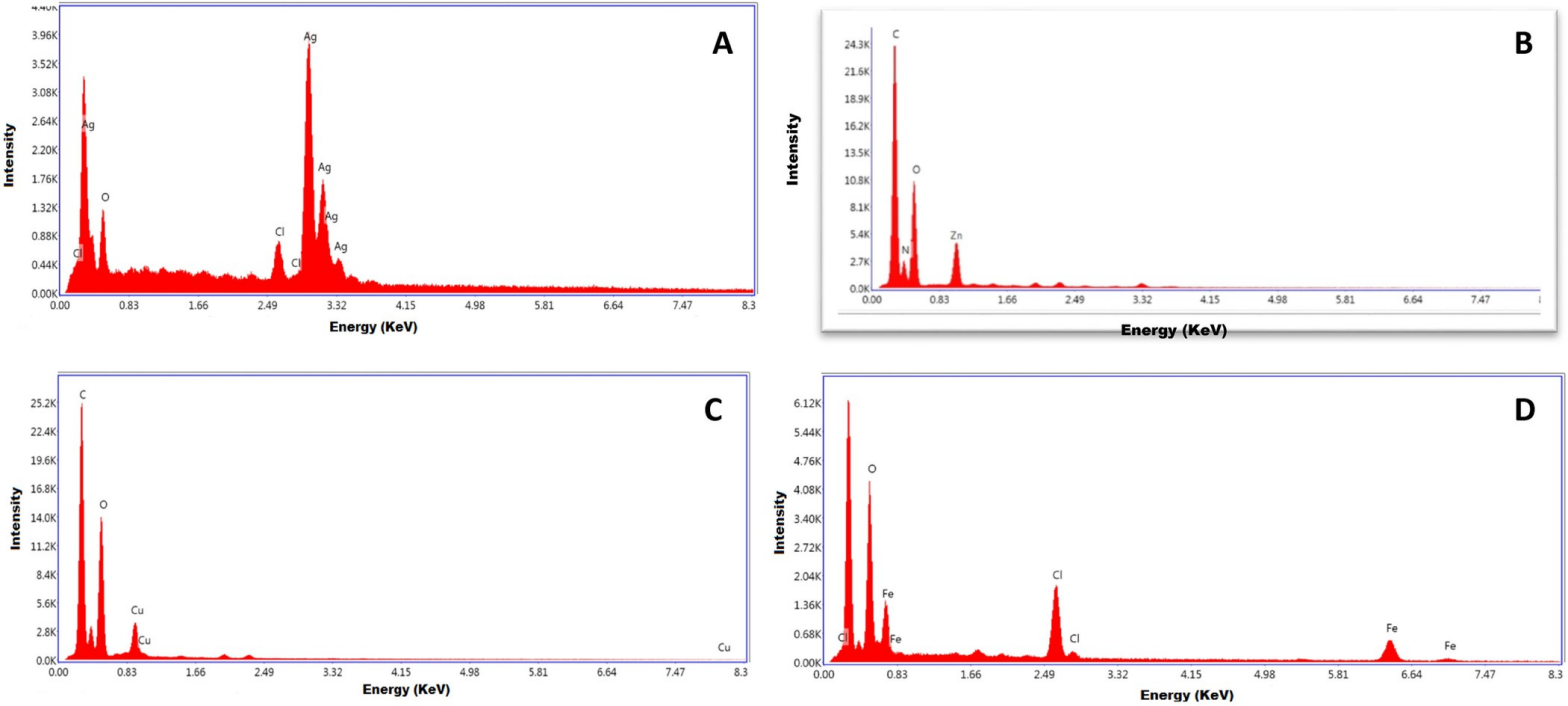
EDX spectrum of A) AgNPs synthesized by using 10 mL (2 mM solution) of AgNO_3_ with 30 mL of *R*. *communis* leaves extract (B) ZnO NPs synthesized by using 10 mL (2 mM solution) of ZnSO_4_ with 30 mL of *R*. *communis* leaves extract (C) CuO NPs synthesized by using 10 mL (2 mM solution) of CuSO_4_ with 30 mL of *R*. *communis* leaves extract (D) FeO NPs synthesized by using 10 mL (2 mM solution) of FeCl_3_ with 30 mL of *R*. *communis* leaves extract.

Qualitative and quantitative identification of the elemental composition of green synthesized metal and metal oxide NPs was examined by EDX analysis. EDX spectrum of AgNPs confirmed the presence of elemental silver (82.62%), along with the signals of Cl and O, as available in the reaction mixture of synthesized AgNPs. It was identified that AgNPs display typical optical absorption peaks in the range 2.7–3.4 keV. Due to the surface plasmon resonance, metallic silver nanocrystals showed an absorption peak approximately at 3 keV. Oxygen and silver were found at a stoichiometric ratio of 15.28: 82.73 which indicates the absence of impurities (Fig 5A).

In the EDX spectrum of ZnO NPs, three peaks between 1 and 10 keV were observed which confirmed the presence of zinc with a strong peak at 1 keV along with peaks of oxygen and carbon (Fig 5B). Zinc peaks could appear from zinc oxide NPs and other peaks may come from organic compounds. Oxygen and zinc were present at a ratio of 20: 80, indicating the absence of impurity. The presence of carbon and oxygen peaks in the EDX spectra might be due to the presence of stabilizing agents from the plant extract which probably acted as a capping agent for the synthesized nanoparticles.

Three prominent peaks were observed between 1 and 10 keV in the EDX pattern of CuO NPs, the peak at 1 keV confirmed the presence of copper (Fig 5C). Peaks of oxygen and carbon were also observed which shows the presence of organic compounds. These peaks were supposed to appear due to stabilizing agents (present in plant extract) which most likely acted as a capping agent for the synthesized NPs. The copper peak could be originated from copper NPs which confirmed the synthesis of NPs. Throughout the scanning range of binding energies, no peak belonging to impurity was detected, which confirmed the high purity of Cu nanoparticles.

EDX study of FeO NPs confirmed the presence of elemental Fe (57.16%), along with the signals of Cl and O. The EDX spectrum of FeO NPs showed intense peaks of Fe and Oxygen and a small peak of Cl. The chlorine signal was originated due to the FeCl_3_ that was used in the NPs synthesis protocol. The peak at 6 keV was observed which confirmed the presence of FeO NPs. The presence of impurities is attributed to the extracellular organic element adsorbed on the surface of the metallic nanoparticles (Fig 5D).

### Fourier transform infrared (FTIR) spectroscopy

FTIR measurements were carried out to identify the possible functional groups present in the plant extract which are responsible for capping and efficient stabilization of the synthesized nanoparticles [51].

The phytochemical evaluation of *R*. *communis* showed the presence of flavonoids, alkaloids, saponins, steroids, and glycosides. The presence of two alkaloids, ricinine (0.55%) and N dimethyl ricinine (0.016%), and six flavones were observed in dried leaves of *R*. *communis* [52].

**Fig 6:**
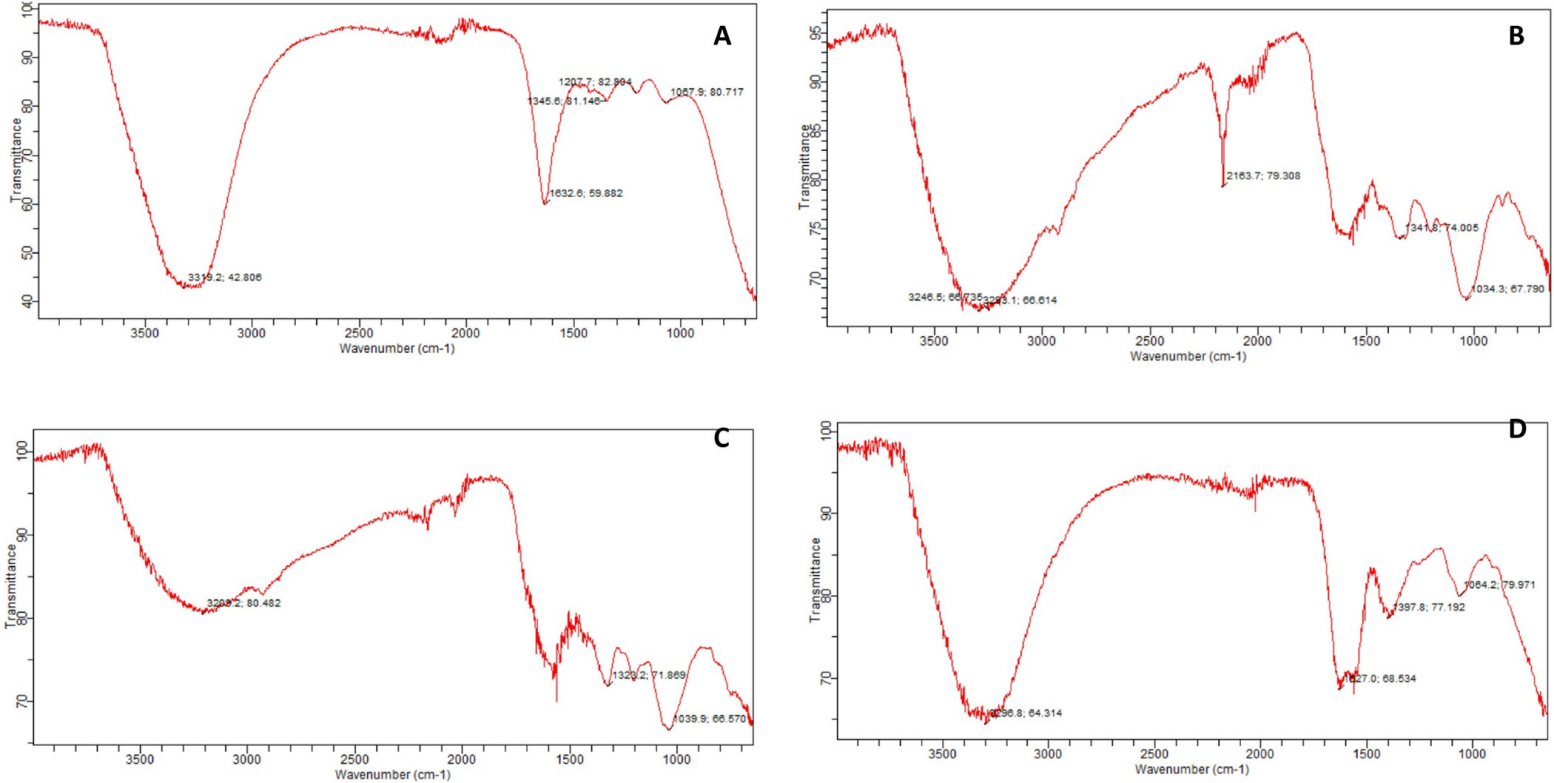
FTIR spectrum of (A) AgNPs prepared by using 10 mL (2 mM solution) of AgNO_3_ with 30 mL of *R*. *communis* leaves extract (B) ZnO NPs prepared by using 10 mL (2 mM solution) of ZnSO_4_ with 30 mL of *R*. *communis* leaves extract (C) CuO NPs prepared by using 10 mL (2 mM solution) of CuSO_4_ with 30 mL of *R*. *communis* leaves extract (D) FeO NPs prepared by using 10 mL (2 mM solution) of FeCl_3_ with 30 mL of *R*. *communis* leaves extract.

FTIR spectrum of synthesized AgNPs is presented in Fig 6A. The FTIR analysis of the AgNPs shows the presence of two strong absorption bands at 3319 cm^−1^ and 1632 cm^−1^. The peak at 3319 cm^−1^ was credited to the hydroxyl functional group of alcohols, polyphenolic or amine whereas the absorption band at 1632 cm^−1^ corresponds to the carbonyl functional group of carboxylic acid or it may be due to the amide I band of proteins. The peak observed at wavelength 1345 cm^-1^ is traceable to the asymmetric stretching vibration of nitrate ions (NO_3_^1^) whereas the absorption band at 1067 cm^−1^ is linked to the vibrations of C–O in C–CCOOR. The absorption peaks observed in the range of 1700–1400 cm^−1^ indicated the presence of AgNPs capped with biomolecules.

Fig 6B is presenting the FTIR spectrum of zinc oxide NPs. The absorption peak observed at 1034 cm^−1^ is attributed to the vibrations of C–O. Absorption bands in the range of 3500 and 2500 cm^−1^ indicated the presence of characteristic OH and N-H stretching of aldehydes. Absorption bands at 700–600 cm^−1^ indicated the formation of zinc oxide nanoparticles. The absorption peaks from 1600–1500 cm^−1^ linked to amide I and amide II regions appearing due to carbonyl stretching in proteins. The appearance of absorption bands at 1400 to 1000 cm^−1^ has been attributed to methylene (from the proteins) in the solution and due to C-N stretching vibrations of amine. The absorption band at wavelength 1341 cm^−1^ credited to the C-C stretching vibration of alcohol, carboxylic acid, ether, and ester. Peaks that appeared at 2500–2000 cm^−1^ indicated the presence of C≡C and C≡N vibrations.

FTIR spectrum of copper oxide nanoparticles observed by the reduction of copper ions using plant extract is presented in Fig 6C. The appearance of a strong peak at 3209 cm^−1^ is characteristic of the hydroxyl functional group of alcohols. The absorption band at 3400 and 2500 cm^-1^ are corresponded to O-H (or N-H) and aldehydic C-H stretching, respectively. Peaks that appeared at 1600 cm^-1^ and 1500 cm^-1^ are linked to amide, which arises due to carbonyl stretching in proteins. C-N stretching vibrations of amine and methylene scissoring vibrations from the proteins were attributed due to bands appeared at 1323 cm^-1^. The appearance of the peak at 1039 cm^−1^ is associated with the vibrations of C–O in C–CCOOR.

The FTIR spectrum obtained from green synthesized FeO NPs is presented in Fig 6D. The appearance of a strong absorption band from 3400 to 3200 cm^−1^ indicates–OH group stretching. Absorption peaks observed from1400 to 1000 cm^−1^ attributed to methylene from the proteins in the solution. Bands that appeared from 1600–1500 cm^−1^ represent the presence of C-N stretching vibrations of amine and due to carbonyl stretching in proteins (amide I and amide II). Frequency appeared at1627 cm^−1^ is credited to C = O stretching. Absorption frequency appeared at 1397 cm^−1^ indicated the presence of ricinoleic acid in the sample. C–O vibrations were observed at 1064 cm^−1^. The absorption band at 630 cm^−1^was attributed to Fe vibrations.

### X-ray diffraction (XRD) analysis

**Fig 7:**
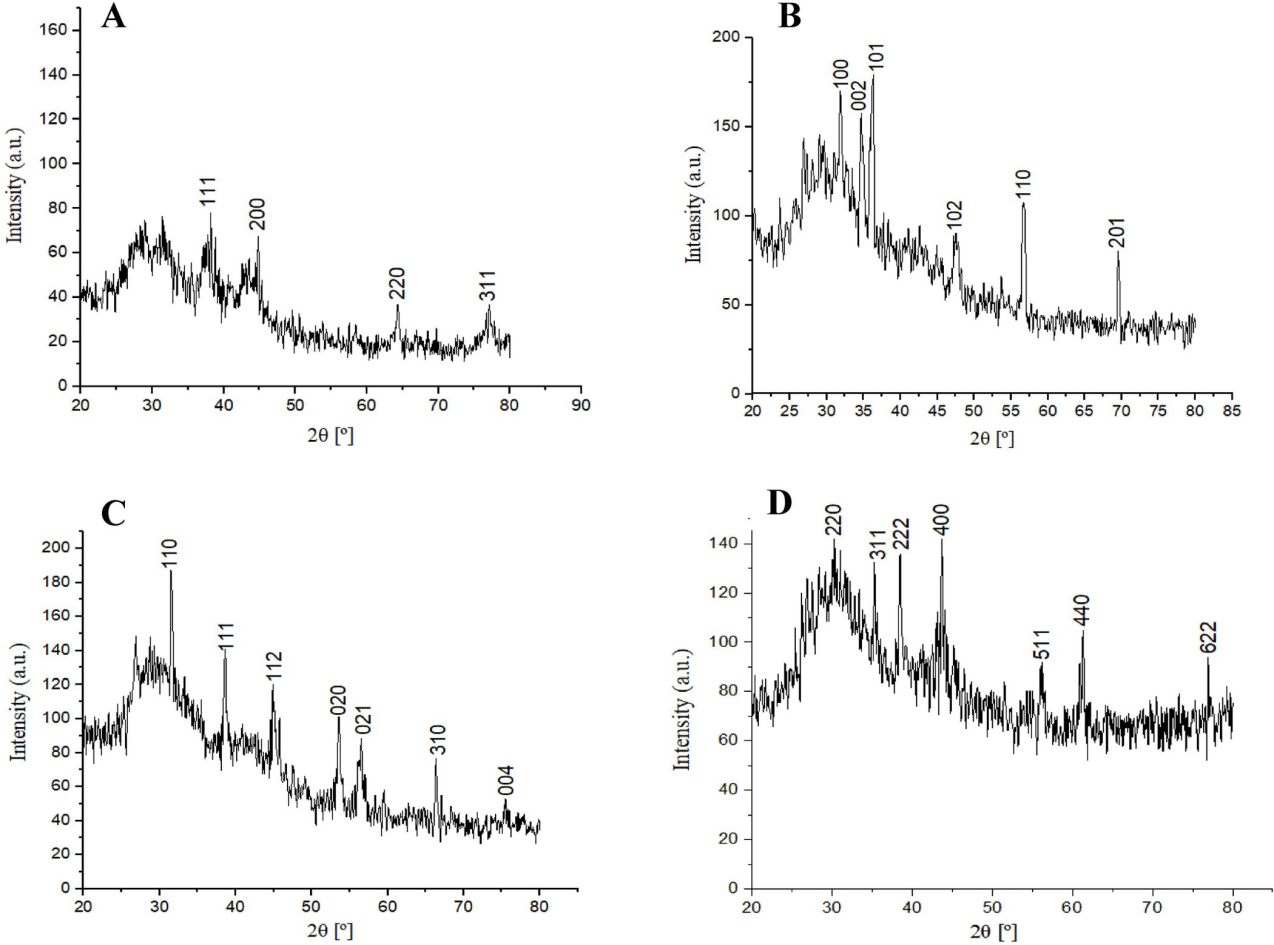
XRD analysis of (A) AgNPs prepared by using 10 mL (2 mM solution) of AgNO_3_ with 30 mL of *R*. *communis* leaves extract (B) ZnO NPs prepared by using 10 mL (2 mM solution) of ZnSO_4_ with 30 mL of *R*. *communis* leaves extract (C) CuO NPs prepared by using 10 mL (2 mM solution) of CuSO_4_ with 30 mL of *R*. *communis* leaves extract (D) FeO NPs prepared by using 10 mL (2 mM solution) of FeCl_3_ with 30 mL of *R*. *communis* leaves extract.

The crystalline nature of the green synthesized NPs was confirmed by using the XRD pattern analysis. The XRD patterns of AgNPs, ZnO NPs, CuO NPs, and FeO NPs are presented in Fig 7A–7D showed several Braggs reflections with various 2θ values. AgNPs exhibited four diffraction peaks at 38.14°, 44.08°, 64.37°, and 77.14° which correspond to the reflection of (111), (200), (220), and (311) planes. X-ray diffraction analysis revealed that AgNPs is a face-centered cubic (FCC) lattice structure in agreement with standard powder diffraction card (JCPDS-ICDD (Joint Committee on Powder Diffraction Standards) files No 04–0783). ZnO NPs presented six distinguished peaks at 2 theta degree 31.8°, 34.7°, 36.1°, 47.4°, 56.6° and 69.5° which corresponds to (100), (002), (101), (102), (110), and (201) planes, respectively. All outstanding diffraction peaks in XRD spectra are compatible with those recorded by JCPDS-ICSD (card No. 89–7102), which confirmed the formation and crystallographic structure of ZnO NPs. For CuO NPs, diffraction patterns were observed at 2θ = 31.5°, 38.6°, 45.1°, 53.5°, 56.5°, 66.4°, and 75.4° were assigned to (110), (111), (112), (020), (021), (310), and (004) crystal planes respectively. Diffraction peaks were observed as the distinctive monoclinic structure CuO NPs. These peaks were in accordance with reference to JCPDS-ICSD file No. 48–1548. The JCPDS-ICSD file No. 019–0629 closely matched with the XRD pattern observed in this study showing the characteristic peaks of FeO NPs at 2θ of 30.0°, 35.2°, 38.5°, 43.7°, 56.2°, 61.3°, and 76.9° corresponding to the face-centered cubic phase of (220), (311), (222), (400), (511), (440) and (622) planes, respectively. The average crystallite size as determined by using the Debye Scherrer equation for AgNPs, ZnO NPs, CuO NPs, and FeO NPs was found to be 7.25, 4.77, 14.6, and 15.3 nm respectively. In the XRD spectrum, some other additional peaks were also seen which were considered to be appeared due to the capping material present in plant extract and may be due to the presence of unreactive metal precursor and other intermediate products.

### Antibacterial activity of green synthesized NPs

An effort has been made in the present research work to examine the antibacterial activity of the green synthesized metal and metal oxide nanoparticles against clinical isolate of gram-positive *S*. *aureus*. Prepared salt solutions, plant extract, and synthesized NPs were evaluated for their antibacterial potential. Very clear zones were observed around the wells loaded with NPs as compared to salt solution and plant extract which proved the effectiveness of NPs.

**Fig 8:**
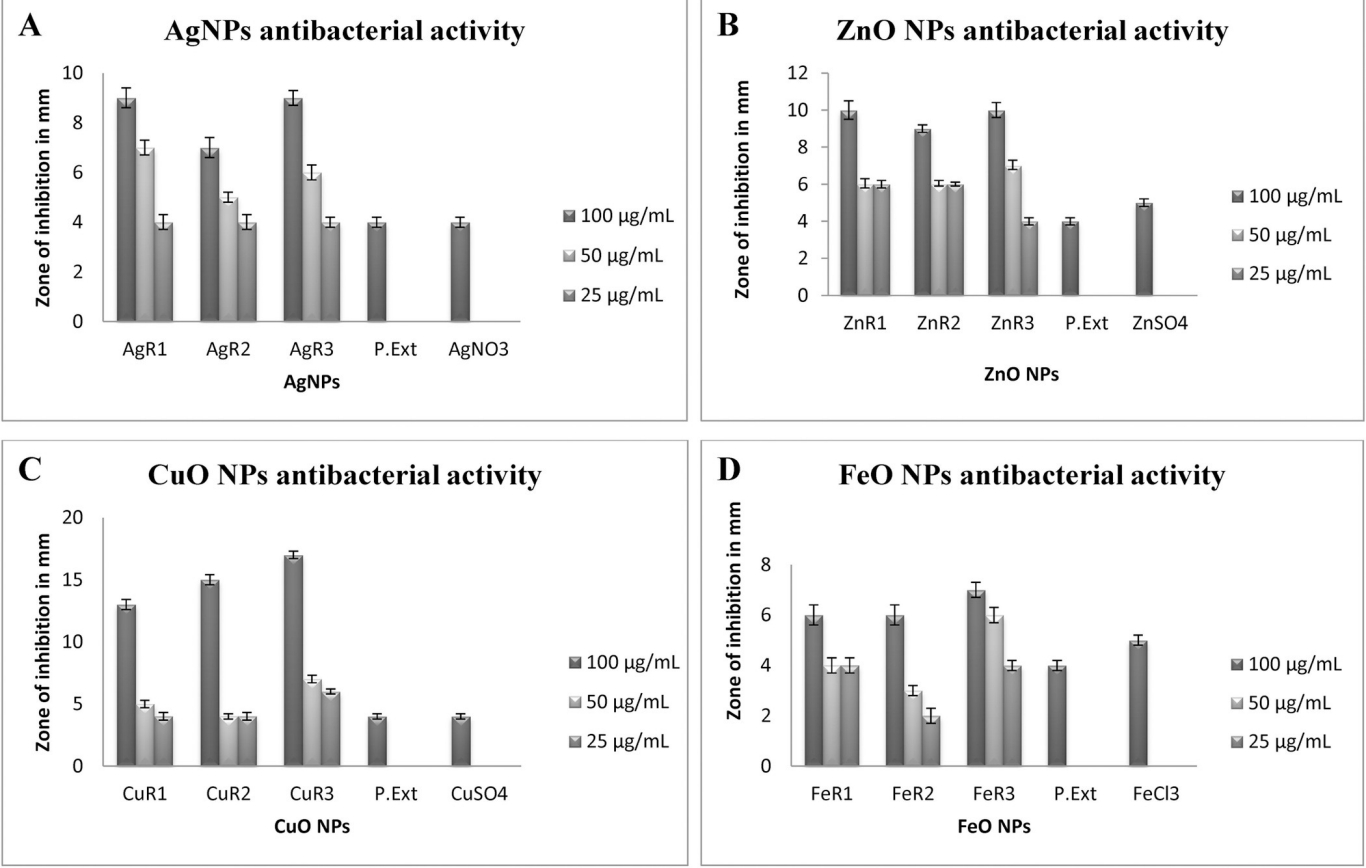
Antibacterial activity of (A) AgNPs synthesized by using 10 mL of 2 mM AgNO_3_ solution with 10, 20 and, 30 mL of *R*. *communis* extract (B) ZnO NPs synthesized by using 10 mL of 2 mM ZnSO_4_ solution with 10, 20 and, 30 mL of *R*. *communis* extract (C) CuO NPs synthesized by using 10 mL of 2 mM CuSO_4_ solution with 10, 20 and, 30 mL of *R*. *communis* extract (D) FeO NPs synthesized by using 10 mL of 2 mM FeCl_3_ solution with 10, 20 and, 30 mL of *R*. *communis* extract.

It was observed that in the case of AgNPs, maximum activity was depicted by AgR_3_ (09 ± 0.5 mm) (Fig 8A). From ZnO NPs, maximum activity was observed in the well loaded with ZnR_3_ (10 ± 0.2 mm) (Fig 8B). In the case of CuO NPs, the highest activity was presented by CuR_3_ (17 ± 0.7 mm) (Fig 8C). In FeO NPs, FeR_3_ provided best antibacterial results (07 ± 0.3 mm) (Fig 8D). It was noted that prominent inhibition zones were observed around the wells loaded with 100 μg/mL of NPs which decreases around the wells containing 50 μg/mL NPs, which further reduced with 25 μg/mL of NPs. The green synthesized metal and metal oxide NPs were found to be dose-dependent (Fig 8A–8D).

Metal and metal oxide nanoparticles significantly affect the cell membrane integrity in both gram-positive and gram-negative bacteria. It is reported by Munoz et al., that different concentrations of nanoparticles depolarized the cell membrane of both gram-positive and gram-negative bacteria that results in increased cell permeability. An increase in membrane permeability facilitates the cellular entrance of NPs, allowing intracellular higher efficiency of NPs [53].

The antimicrobial effect of NPs may be due to the multifaceted mechanism by which nanoparticles interact with the bacteria. Nanoparticles affect the metabolic process of bacterial cells by several multi-level modes of action. NPs can easily bind to bacterial cell surfaces because of electrostatic attraction between the negatively charged cell membrane of microorganisms and the positive surface charge of the NPs. Metal ions interact with the sulfur-containing proteins present in the bacterial cell wall which causes damage to the cell wall of bacteria [54]. It leads to an increase in permeability which ultimately disrupts the phosphate ion and potassium ion pump which becomes the cause of outflow of cellular content which ultimately causes cell disintegration. It is also reported that when NPs are dissolved in water or when they penetrate the cells, they released a definite amount of cations. The antibacterial potential is credited to both physical properties and elution of ions of NPs. NPs and free ions both play a significant role in produce a strong antibacterial activity.

It is reported earlier that metal oxide NPs slowly release metal ions that are taken up by the cell. These metal ions moved in the intracellular compartment and interact with functional groups of proteins and nucleic acids like carboxyl (–COOH), mercapto (–SH), and amino (–NH) groups. After the interaction, it alters the structure of the cell, hampers the enzymatic activity, and disturbs the normal physiological processes in the bacterial cell leading to cell death.

It is also studied by Daming and colleagues that NPs can easily enter the microbial body and damages its intracellular structures. It may denature the ribosome and causes inhibition of protein synthesis. It may also block the translation and transcription process by binding with the genetic material of the bacterial cell [55].

It is also reported by Rudramurthy et al., 2016 that NPs are responsible for the increased cellular oxidative stress in microbes which is associated with the antibacterial potential of the particles. ROS (reactive oxygen species) are natural by-products of cellular oxidative metabolism and have significant importance in cell survival and cell death. The imbalance in the concentration of reactive oxygen species (ROS) and free radicals causes oxidative stress. It may also consider as increased concentration of these moieties becomes the cause of a toxic effect. The increase in the production of ROS may also disturb redox homeostasis causing oxidative stress which affects the membrane lipids and alters the structure of protein and DNA [56].

### Evaluation of the synergistic effect of Streptomycin with NPs against *S*. *aureus*

**Fig 9:**
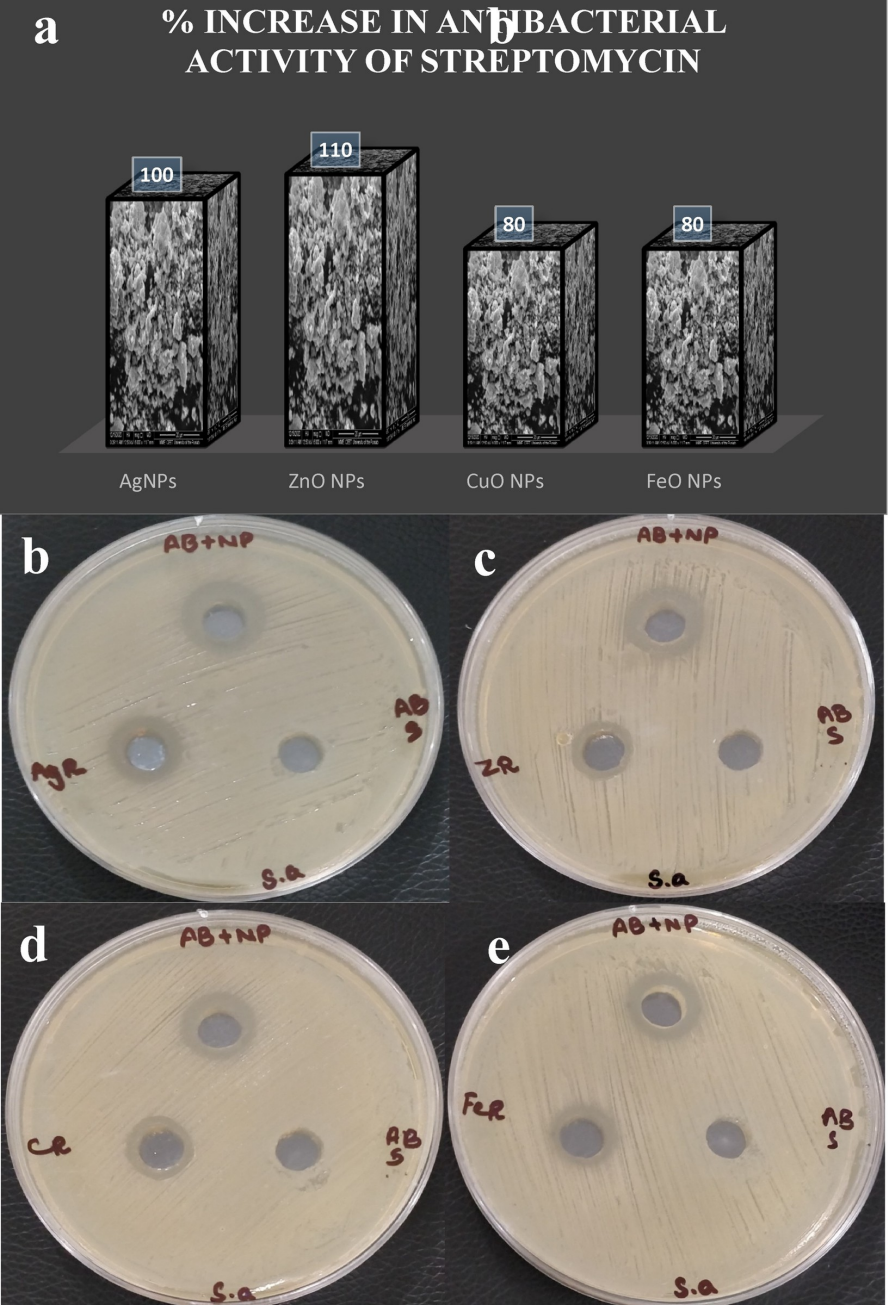
Synergistic effect of combine formulation of (B) AgNPs-Stp (C) ZnO NPs-Stp (D) CuO NPs-Stp (E) FeO NPs-Stp against *S*. *aureus*.

Once the antibacterial activity was determined for all NPs, the question arises whether combining green synthesized metal and metal oxide nanoparticles with commercially used antibiotics enhances the antibacterial activity or not. It is found true from the present investigation that streptomycin when combined with nanoparticles showed enhanced antibacterial activity against *S*. *aureus* (Table 2). The figures are also displaying the increased zone of inhibition area after combining nanoparticles with antibiotic (Fig 9B–9E).

**Table 2:** Antibacterial activity of Streptomycin before and after conjugation with green synthesized NPs against *S*. *aureus*.

**Antibiotic**	**Inhibition zones in mm**				
	**Antibiotic alone (a)**	**AgNPs (b)**		**ZnO NPs (c)**	
		**AB + AgNPs (b)**	**% Fold increase = ((b-a)/a)×100**	**AB + ZnO NPs (c)**	**% Fold increase = ((c-a)/a)×100**
**Streptomycin**	00 ± 0.0	10 ± 0.5	100	11 ± 0.5	110
		**CuO NPs (d)**		**FeO NPs (e)**	
		**AB + CuO NPs (d)**	**% Fold increase = ((d-a)/a)×100**	**AB + FeO NPs (e)**	**% Fold increase = ((e-a)/a)×100**
		08 ± 0.2	80	08 ± 0.5	80

Concentration expressed in μg/mL.

Each value is the representative mean of three replicates. Standard deviations did not exceed 5%.

It was noticed that Streptomycin alone was inactive against *S*. *aureus* while wells inoculated with conjugates (streptomycin loaded NPs) showed significantly higher inhibition zones in all the plates which made it clear that there is some synergistic effect. Inhibition zones presented by AgNPs-Stp, ZnO NPs-Stp, CuO NPs-Stp and FeO NPs-Stp were 10 ± 0.5 mm, 11 ± 0.5 mm, 08 ± 0.2 mm and 08 ± 0.5 mm respectively (Fig 9B–9E).

Results showed that the antibacterial action of streptomycin with NPs against *S*. *aureus* was significantly increased as antibiotic alone depicted no activity. Interestingly, the free antibiotic which failed to inhibit the growth of bacteria has become highly antibacterial when combined with nanoparticles. Combinations of streptomycin with all four green nanoparticles showed a synergistic effect against *S*. *aureus*. Among all the four nanoparticles combined with streptomycin, ZnO NPs-Stp showed the highest antibacterial potential and 110% fold increase in the antibacterial activity (Fig 9C). Whereas combined formulations of silver (AgNP-Stp) copper (CuO NP-Stp) and iron (FeO NPs-Stp) NPs with streptomycin showed 100%, 80%, and 80% fold increase in the antibacterial activity respectively against *S*. *aureus* (Fig 9A).

The mechanism of conjugate formation by using nanoparticles and antibiotic is not exactly known but researchers presented two assumptions [57,58]. Firstly, it is assumed that conjugate formation was facilitated by a large surface area to volume ratio of NPs to antibiotic molecules; so the antibiotic can easily bind to the PBP (Penicillin-binding proteins-membrane associated proteins) receptor of the bacterial membrane. It assists the antibiotic in accumulation on the outer surface of the bacterial membrane [57]. It infringes the osmotic balance which disrupts the cell membrane and inhibition of bacterial cell wall synthesis [59]. It leads to leakage of cytoplasmic contents which causes cell death.

Secondly, antibiotic-NPs conjugate targets many sites in the bacterial cell rather than binding and penetrating the cell wall [60]. When conjugates go through in bacterial cell, interrupt the regulatory functions, deactivate the bioactive proteins, stop the sulfur-phosphorous interaction with nucleic acids which leads to inhibition of protein synthesis [57].

Because of transport scarcity, many of the available antibiotics usually do not reach high intracellular concentrations and this condition gets worse in the presence of resistance mechanisms, like increased efflux and decreased uptake of antibiotic. NPs efficiency depends upon the high surface-area-to-volume ratio. Percentage of atoms at the surface and surface forces become more dominant when the surface area-to-volume ratio increases. Nanoparticles bind to proteins in the bacterial cell membrane which causes an increase in permeability that leads to an increase in antibiotic infiltration into the bacterial cell. Therefore, nanoparticles in conjugation with antibiotics enhance the amount of antimicrobials at specific sites of the cell membrane [61,62]. Due to the electrostatic attraction between the negatively charged cell membrane of microorganisms and the positive surface charge of the NPs, NPs can bind easily to the bacterial cell surface (however, not all NPs are positively charged). Interaction between sulfur-containing proteins in the bacterial cell wall and metal ions also disrupts the cell wall [54]. NPs can easily interfere with essential microbial metabolic pathways because of their nano size; they can interact with bacteria and can go through both bacterial envelopes and the host’s cell membranes effortlessly [63].

This synergistic effect is also size dependent, but not much related to shape of nanoparticles as it is clear from present study. Silver and copper nanoparticles are both spherical while zinc oxide and iron oxide nanoparticles, both are found to be hexagonal and triangular. Whereas their synergistic activity with streptomycin is totally opposite. But their synergistic effect can be related to their size as AgNPs and ZnO NPs with minimum size (7.25 and 4.77 nm) gave maximum synergistic effect with streptomycin i.e., 100 and 110% respectively.

Li et al., 2005 explained the synergistic effect of AgNPs and amoxicillin. They observed that in the AgNPs-amoxicillin complex, each NP is bounded by many amoxicillin molecules between the hydroxyl and amino groups of amoxicillin [64]. Afterward, Duran et al. pointed out another important binding the sulfur bridge between amoxicillin and NPs. A combination of both assists each other in penetration and effective action in microorganisms [65].

It is also reported by Roshmi et al., 2015 that gold nanoparticles can be used as carriers for antibiotics that adhere to the surface via electrostatic attraction [66]. Some researchers observed that gold NPs in conjugation with antibiotics can be used for enhancing the solubility of poorly water-soluble drugs. AuNPs-ATB improves drug half-life and systemic circulation time and stimulates the continuous responsive drug release [67,68].

It was also explained by some researchers that increased localization of drug on the bacterial surface increased the bacteria-antibiotic interactions that facilitate the binding of antibiotics to bacteria, which becomes the basis of enhanced activity of the conjugate and block bacterial efflux pumps [34,60,69].

With some key points in mind from the results, it can be suggested that nanoparticles damage the cell wall of the bacteria and facilitate the entrance of antibiotic into the bacterial cell. Nanoparticles in conjugation with antibiotics increase the concentration of antimicrobials at a specific site of the cell membrane and disrupt the metabolic pathways, interrupt the protein-synthesizing machinery which leads to cell death.

### Minimum inhibitory concentration (MIC) of conjugates against *S*. *aureus*

After confirmation of antimicrobial activity of AgNPs-Stp, ZnO NPs-Stp, CuO NPs-Stp, and FeO NPs-Stp through well diffusion assay, minimum inhibitory concentration (MIC) against *S*. *aureus* was also determined. MIC of all the conjugates was examined carefully and MIC of AgNPs-Stp, ZnO NPs-Stp, CuO NPs-Stp, and FeO NPs-Stp was observed to be 3.12, 2.5,10, and 12.5 μg/mL respectively against *S*. *aureus* (Table 3).

**Table 3:** Determinations of minimum inhibitory concentration (μg/mL) of prepared conjugates against *S*. *aureus*.

MIC of conjugates (μg/mL)				
**Conjugates**	AgNPs-Stp	ZnO NPs-Stp	CuO NPs-Stp	FeO NPs-Stp
**MIC**	3.12±0.9	2.5±0.11	10±0.93	12.5±0.50

Results are the mean of at least three different experiments. Standard deviations did not exceed 5%.

Concentration expressed in μg/mL.

The maximum amount of sugars and proteins was released by *S*. *aureus* (120.75 and 13.5 μg/mL, respectively) when treated with ZnO NPs-Stp. The leakage of sugars and proteins depends upon membrane permeability. Enhanced permeability of the membrane due to NPs leads to leakage of proteins and sugars.

Combine treatment of antibiotics in conjugation with nanoparticles provides possibilities for the usage of antibiotics that have fallen into disuse because of bacterial resistance. It can provide additional potential for the treatment of healthcare, veterinary, and agriculture sectors. Further studies and research is still urgently needed for predicting a more feasible and efficient design to develop a novel antimicrobial drug to combat multidrug-resistant bacteria.

## Conclusion

The potential of *R*. *communis* to reduce metal ions into metal nanoparticles such as AgNPs, ZnO NPs, CuO NPs, and FeO NPs has been evaluated. An attempt has been made in the present investigation to examine the antibacterial potential of AgNPs, ZnO NPs, CuO NPs, and FeO NPs, free antibiotic and conjugates of antibiotic (streptomycin) with NPs against *S*. *aureus*. Nanoparticles depict a pronounced antibacterial activity against *S*. *aureus*. The synergistic effect was observed when streptomycin was used in combination with NPs. Streptomycin in combination with ZnO NPs-Stp showed the highest synergistic effect against *S*. *aureus*. A gram-positive bacterium (*S*. *aureus*) that was resistant to streptomycin becomes susceptible to the same antibiotic in combination with nanoparticles. Synergistic antibacterial properties of nanoparticles with antibiotics provide an alternative approach to minimize drug resistance and provide potential applications in the medical field. Biogenic metallic and metal oxide nanoparticles may be used effectively in combating MDRS.
